# Arp2/3-Branched Actin Maintains an Active Pool of GTP-RhoA and Controls RhoA Abundance

**DOI:** 10.3390/cells8101264

**Published:** 2019-10-16

**Authors:** Yuxing Huang, Xin Yi, Chenlu Kang, Congying Wu

**Affiliations:** Institute of Systems Biomedicine, Beijing Key Laboratory of Tumor Systems Biology, School of Basic Medical Sciences, Peking University Health Science Center, Peking University, Beijing 100191, China; yxhuang1898@pku.edu.cn (Y.H.); jxyx0728@163.com (X.Y.); wushukcl@163.com (C.K.)

**Keywords:** Arp2/3 complex, branched actin, RhoA, ubiquitination, Smurf1, cytokinesis

## Abstract

Small GTPases regulate cytoskeletal dynamics, cell motility, and division under precise spatiotemporal control. Different small GTPases exhibit cross talks to exert feedback response or to act in concert during signal transduction. However, whether and how specific cytoskeletal components’ feedback to upstream signaling factors remains largely elusive. Here, we report an intriguing finding that disruption of the Arp2/3-branched actin specifically reduces RhoA activity but upregulates its total protein abundance. We further dissect the mechanisms underlying these circumstances and identify the altered cortactin/p190RhoGAP interaction and weakened CCM2/Smurf1 binding to be involved in GTP-RhoA reduction and total RhoA increase, respectively. Moreover, we find that cytokinesis defects induced by Arp2/3 inhibition can be rescued by activating RhoA. Our study reveals an intricate feedback from the actin cytoskeleton to the small GTPase. Our work highlights the role of Arp2/3-branched actin in signal transduction aside from its function in serving as critical cytoskeletal components to maintain cell morphology and motility.

## 1. Introduction

Small GTPases have long been identified to act as upstream regulators for the actin cytoskeleton [1]. Rac1 promotes lamellipodia formation by activating the Arp2/3 complex and by inducing branched actin formation [2], while Cdc42 is well known to induce filopodial protrusion and invadopodium formation by formin Dia2 and FMNL2 [3,4]. The cooperation of Rac1 and Cdc42 is essential in generating cell polarity during cell migration [5]. RhoA interacts with downstream effectors to influence actin stability and to generate actomyosin contraction [6]. The binding of mDia1 to active RhoA (GTP-RhoA) promotes linear actin formation [7,8]. A ring of RhoA has been observed to localize to the site of the nascent cytokinetic furrow in early cytokinesis and facilitates the formation of cytokinetic furrow [9]. Active RhoA enhances contractile ring assembly through its downstream effectors including the Rho kinase (ROCK) [10]. ROCK phosphorylates and activates LIM kinase, which in turn inhibits the actin-severing protein cofilin, resulting in stabilization of the actin network [11].

RhoA is activated by guanine–nucleotide exchange factors (GEFs) and inactivated by GTPase activating proteins (GAPs) [12]. p190RhoGAP is a major negative regulator of Rho GTPases and is expressed in cells as two isoforms that share a common GTP-binding domain and that controls RhoA activity: p190ARhoGAP (also known as ARHGAP35) and p190BRhoGAP (also known as ARHGAP5) [13,14]. p190ARhoGAP contains a protrusion localization sequence (PLS), which is necessary for p190ARhoGAP targeting to the leading edge [15]. PLS deletion or mutation (S866F and delta 865–870) dramatically enhances p190ARhoGAP activity toward RhoA [14]. The cortical actin-binding protein cortactin, which is a weak nucleator of the Arp2/3 complex, acts as a scaffold for p190ARhoGAP and is also required for leading edge localization of p190ARhoGAP [15]. The binding of cortactin to PLS of p190ARhoGAP results in a closed and inactivated conformation of p190ARhoGAP, while its dissociation leads to opening of p190ARhoGAP and locally turns off RhoA [14]. Aside from the activity, protein abundance also affects cellular functions of small GTPases. The proteasomal degradation is the main pathway for the degradation of RhoA [16]. Cullin3, SCF^FBXL19^, and Smurf1 have been reported to be E3 ubiquitin ligases critical for proteasome-dependent RhoA degradation [16,17]. ERK2 is required for SCF^FBXL19^-mediated RhoA ubiquitination [16], while Cerebral Cavernous Malformation 2 (CCM2) has been demonstrated to act as an important scaffolding protein in facilitating Smurf1-mediated RhoA degradation [18,19].

Cross talks between different small GTPases has been observed. Rac1 has been reported to bind and activate p190RhoGAP, resulting in RhoA inhibition [20]. Conversely, RhoA inhibits Rac1 activity by activating the Rac1 GAP FilGAP through ROCK [21]. However, surprisingly scarce knowledge in the feedback regulation from the cytoskeleton to the small GTPases has been obtained [22], which includes positive regulation of RacGEFs by F-actin through recruitment of PI3K to the plasma membrane [23]. Precise spatiotemporal control of cell morphology and motility rely on feedback regulations, and such mechanisms involving downstream cytoskeleton in affecting upstream signaling factors have been largely understudied. Here, we report an intriguing finding that disruption of the Arp2/3-branched actin specifically reduces RhoA activity through altered cortactin/p190RhoGAP interaction but upregulates RhoA abundance by affecting CCM2/Smurf1. Moreover, we found that cytokinesis defects induced by Arp2/3 inhibition can be rescued by activating RhoA, demonstrating the physiological significance of this cytoskeleton–small GTPase signaling feedback loop. Our work sheds new lights on branched actin, highlighting its role in signal transduction aside from serving as critical cytoskeletal components to maintain cell morphology and motility.

## 2. Materials and Methods

### 2.1. Cell Lines and Cell Culture

HEK293T, Mouse embryonic fibroblasts (MEFs), and Hela cells were kept by our laboratory. All the cells were passed no more than 10 times and were mycoplasma free. Cells were cultured in Dulbecco’s Modified Eagle Medium (DMEM) media supplemented with 10% fetal bovine serum (FBS) (PAN, Biotech, P30-3031, Aidenbach, Germany), 100 U/mL penicillin, and 100 µg/mL streptomycin at 37 °C with 5% CO_2_.

### 2.2. p190RhoGAP Knockout and CCM2 and ARHGAP29 Knockdown Cell-Line Generation

The following short hairpin RNA (shRNA) and primers were used to generate CCM2 knockdown HEK293T:

Human shRNA forward shRNA-1, 5′-GCCCAGGTCCTCTACTGTG-3′.

Human shRNA forward shRNA-2, 5′-GCTGAGCGACTATATTGAG-3′.

The following shRNA and primers were used to generate ARHGAP29 knockdown Hela:

Human shRNA forward shRNA-1, 5′-GCATCAGGTCAACTCTCTACT-3′.

Human shRNA forward shRNA-2, 5′-GGGCTCAAGTCCTTAAGTTCC-3′.

The following small guide RNA (sgRNA) and primers were used to generate p190RhoGAP knockout Hela:

Human sgRNA forward sgRNA-1, 5′-CACCGATTGAGTACATTGAAGCCAC-3′.

Human sgRNA forward sgRNA-2, 5′-CACCGTAACAACTGTCGTGACTCCA-3′.

Human sgRNA forward sgRNA-3, 5′-CTTCTTGACATTCTTTCTAGCAGTT-3′.

A single cell clone from lentivirus-infected pool cells was selected and verified by western blotting and DNA sequencing.

### 2.3. Western Blot

For western blotting, cells were washed with dulbecco’s phosphate buffered saline (DPBS) once and lysed in an appropriate volume of radio immunoprecipitation assay (RIPA) buffer (50 mM Tris-HCl, pH 8.0, 150 mM NaCl, 1% Triton X-100, 0.5% Na-deoxycholate, 0.1% SDS, 1 mM EDTA, and protease inhibitor cocktail) for 10 min on ice. Lysates were centrifuged at 12,000 rpm for 10 min, and the supernatants were collected. Five times SDS loading buffer was added to the supernatants and boiled for 10 min at 95 °C. Protein samples were run on 10% SDS PAGE acrylamide gels [H_2_O, 30% acrylamide, 1.5 M Tris-HCl pH 8.8, 10% ammonium persulphate, and tetramethylethylenediamine (TEMED)] and transferred onto nitrocellulose membranes by wet electrophoretic transfer, followed by 10% nonfat-milk blocking at 4 °C overnight or at room temperature for 1 h. The first and second antibody incubations were at 4 °C overnight or at room temperature for 2 h. The following antibodies were used: anti-GAPDH (Abcam ab181602, 1:5000, Cambridge, MA, USA), anti-RhoA (Cytoskeleton ARH04, 1:500, Denver, CO, USA), anti-p190RhoGAP (BD Transduction Laboratories 610150, 1:1000, San Jose, CA, USA), anti-HA tag (M180-3, 1:1000), anti-Rac1 (Cytoskeleton ARC03, 1:500, Denver, CO, USA), anti-Cdc42 (Cytoskeleton ACD03, 1:500, Denver, CO, USA), anti-YAP (Cell Signaling Technology 14074s, 1:500, Danvers, MA, USA), anti-ARHGAP29 (Santa Cruz Biotechnology sc-377022, 1:1000), anti-CCM2 (Sigma SAB1400724, 1:500, Saint Louis, MO, USA), anti-pMLC (Cell Signaling Technology 3674s, 1:1000, Danvers, MA, USA), anti-lamin B (proteintech 66095-I-Ig, 1:2000, Rosemont, IL, USA), anti-HA tag (MBL 561, 1:1000, Woburn, MA, USA), anti-DDDDK-tag (MBL M185-3L, 1:1000, Woburn, MA, USA), anti-mouse (sc-2005, 1:5000), and anti-rabbit (sc-2004, 1:5000) Horseradish Peroxidase (HRP) -conjugated secondary antibodies from Santa Cruz. Immobilon Western chemilum HRP substrate (WBKLS0050, Millipore, Burlington, MA, USA) was used. Solution A and solution B were mixed at the ratio of 1:1; then, the mix was added onto nitrocellulose filter membrane. X-ray films were used to detect and record the band intensity. The fixed X-ray films were then scanned. The images were quantified by the Image J software (https://imagej.nih.gov/ij/, image J→Analyze→Gels). The intensity was normalized to GAPDH.

### 2.4. Drug Treatment

The Arp2/3 complex inhibitor CK-666 (Sigma-Aldrich, 182515, Saint Louis, MO, USA) was used at the concentration of 100 μM for 5–6 h. SMIFH2 (Sigma-Aldrich, S4826, Saint Louis, MO, USA) was used to inhibit formin-induced linear actin at the concentration of 15 μM for 5–6 h. Rho activator II (Cytoskeleton CN03, Denver, CO, USA) was used at the concentration of 2 μg/mL or 4 μg/mL for 5 h. The proteasome inhibitor MG-132 (Millipore, 474790-20MG, Burlington, MA, USA) was used at the concentration of 20 μM. Cycloheximide (Sigma-Aldrich, C7698, Saint Louis, MO, USA) was used at the concentration of 10 μg/mL.

### 2.5. Active RhoA/Rac1/Cdc42 Pulldown Assay

Cells were lysed in RIPA buffer (50 mM Tris-HCl, pH 8.0, 150 mM NaCl, 1% Triton X-100, 0.5% Na-deoxycholate, 0.1% SDS, 1 mM EDTA, and protease inhibitor cocktail) for 10 min on ice. Lysates were centrifuged at 12,000 rpm for 10 min, and the supernatants were collected. Twenty μg Rhotekin-RBD Protein GST Beads (Cytoskeleton RT02, Denver, CO, USA) or PAK-GST Protein Beads (Cytoskeleton PAK02, Denver, CO, USA) were washed three times using RIPA buffer (50 mM Tris-HCl, pH 8.0, 150 mM NaCl, 1% Triton X-100, 0.5% Na-deoxycholate, 0.1% SDS, 1 mM EDTA, and protease inhibitor cocktail). Cell lysates (800 μg) were incubated with the beads at 4 °C for 1 h. After incubation, the beads were washed three times using wash buffer (25 mM Tris pH 7.5, 30 mM MgCl_2_, and 40 mM NaCl). Two times loading buffer (100 mM Tris-HCl, pH 6.8, 200 mM dithiothreitol (DTT), 4% SDS, 0.2% bromophenol blue, and 20% glycerol) were added to suspend the beads and boiled for 10 min at 95 °C. Active RhoA, active Rac1, or active Cdc42 was then detected by western blot by using anti-RhoA (Cytoskeleton ARH04, Denver, CO, USA), anti-Rac1 (Cytoskeleton ARC03, Denver, CO, USA), or anti-Cdc42 (Cytoskeleton ACD03, Denver, CO, USA).

### 2.6. Active p190RhoGAP Pulldown Assay

Fifty μL GST resins (Senhui Microsphere, SH-12-1605-13, Suzhou, China) were washed three times using binding buffer [10 mM N-2-hydroxyethylpiperazine-N-ethane-sulphonicacid (HEPES), pH 7.6, 60 mM NaCl, 1 mM MgCl2, 10 mM DTT, 10% glycerol, and 0.01% tween-20], 8 μM RhoA Q63L, and 50 μL GST resins in 200 μL binding buffer were incubated at 4 °C for 1 h. Cells were lysed in lysis buffer [150 mM NaCl, 20 mM HEPES, pH 7.6, 10 mM MgCl_2_, 1% Triton X-100, 1 mM Phenylmethylsulfonyl fluoride (PMSF), and 10 μg/mL aprotinin and leupeptin]. Cell lysates were incubated with 50 μg/mL glutathione Sepharose-bound GST-RhoA^Q63L^ for 1 h at 4 °C. The beads were washed three times with lysis buffer and then boiled at 95 °C for 5 min. Anti-p190RhoGAP (BD Transduction Laboratories 610150, 1:1000, San Jose, CA, USA) was used to test active p190RhoGAP by western blot.

### 2.7. Proximity Ligation Assay (PLA)

To detect the interaction between p190RhoGAP with cortactin in vivo, the proximity ligation assay was performed with Duolink kits from Sigma-Aldrich (DUO92013). Cells were treated with CK-666 (Sigma-Aldrich, 182515, Saint Louis, MO, USA), followed by fixation with 4% paraformaldehyde at room temperature for 10 min. Then, the cells were permeabilized with 0.5% Triton X-100 in phosphate buffer saline (PBS) for 5 min, followed by 3 times wash with PBS. Commercial blocking solution (Sigma-Aldrich, DUO92013, Saint Louis, MO, USA) was added to the samples to incubate for 1 h at room temperature. Mouse anti-p190RhoGAP (1:25) was mixed with rabbit anti-Cortactin antibodies and diluted (1:200) in the antibody diluent. After removing the blocking solution, the diluted antibodies were incubated with cells for 1 h at room temperature followed by 3 × 5-min wash in PBS. The PLUS and MINUS PLA probes were mixed and diluted (1:5) in antibody diluent and incubated with samples for 1 h at 37 °C. Then, the samples were washed in 1× wash buffer A for 2 × 5 min. The ligase was diluted (1:40) in diluted ligation buffer (1:5 in H_2_O), and the mixture was incubated with samples for 1 h at 37 °C. After washing the samples in 1× wash buffer A for 2 × 2 min, the polymerase was diluted (1:80) in diluted amplification stock (1:5 in H_2_O), and the mix was incubated with samples for 100 min at 37 °C. The samples were then washed in 1× wash buffer B for 2 × 10 min followed by another wash in 0.01× wash buffer B for 1 min. Finally, the samples were mounted with Prolong Diamond Antifade with 4′,6-diamidino-2-phenylindole (DAPI) (Life Technologies, P36962, Carlsbad, CA, USA) for 30 min at room temperature.

### 2.8. Immunofluorescence and Imaging Analysis

Cells were plated on acid-washed coverslips overnight. Cells were then fixed with 4% paraformaldehyde (PFA) at room temperature for 15 min, permeabilized in 0.5% Triton X-100 in PBS for 10 min, washed with PBS once for 5 min, and blocked with 5% bovine serum albumin (BSA) for 1 h. For p-MLC (Cell Signaling Technology 3674s, 1:200, Danvers, MA, USA) and p190RhoGAP (1:100) staining, antibodies were diluted 1:200 in 1% BSA (Sigma, Saint Louis, MO, USA) and incubated for 1 h at room temperature. After 3 times washing with PBS, the coverslips were incubated with Alexa Fluor 488 secondary antibody (A-21202, Life Technologies, Carlsbad, CA, USA) and Acti-stain 488 phalloidin (PHDR1-A, Cytoskeleton, Denver, CO, USA) or Rhodamine Phalloidin (PHDR1, Cytoskeleton, Denver, CO, USA) for 0.5 h at room temperature. After another 3 times PBS washing, the coverslips were mounted with Prolong Diamond Antifade with DAPI. Images were captured using a spinning disk microscope (Dragonfly, Andor, Belfast, UK).

### 2.9. Co-IP Assay

HEK293T was plated on 10-cm dishes and transfected with different plasmids. Cells were treated with DMSO or CK-666 48 h before being washed with PBS and lysed with pierce lysis buffer (25 mM Tris-HCl pH 7.4, 150 mM NaCl, 1% NP-40, 1 mM EDTA, and 5% glycerol). Added into the cell lysate was 0.2–2 μg of anti-DDDDK-tag (MBL M185-3L, Woburn, MA, USA) antibody for 4 °C overnight incubation, and 20 μL protein A + protein G agarose (Beyotime, P2012, Shanghai, China) were added to the lysate and incubated for 3 h at 4 °C. Agarose beads were washed with pierce lysis buffer for 3 times and centrifuged at 2500 rpm for 5 min, and 30–40 μL 2× SDS-PAGE loading buffer was added into the beads and boiled for 5 min. Anti-HA tag (MBL 561, 1: 1000, Woburn, MA, USA) and anti-DDDDK-tag (MBL M185-3L, 1:1000, Woburn, MA, USA) were used for detection by western blot.

### 2.10. Live-Cell Imaging

RhoA biosensor live-cell imaging was acquired with an oil objective lens (NA 1.4, Olympus, Tokyo, Japan) on a total internal reflection fluorescent (TIRF) microscope (CellTIRF, Olympus, Tokyo, Japan). Cells were plated on glass-bottom cell-culture dish before imaging. For RhoA biosensor imaging, the interval was 10 min. Cells were maintained in DMEM media supplemented with 10% FBS (PAN, Biotech, Aidenbach, Germany), 100 U/mL penicillin, and 100 µg/mL streptomycin at 37 °C throughout the imaging process. Images were acquired for 6 h. For cytokinesis live-cell imaging, MEFs were plated onto 30-mm cell-culture dish (Corning, Corning, NY, USA), and images were acquired using a 10× dry objective lens (NA 0.3, Olympus, Tokyo, Japan) on a deconvolution microscope (CellTIRF). Single-plane multipoint acquisitions were captured every 5 min within a 37 °C chamber.

### 2.11. Cell Contractility Assay

Collagen was added into gel mix (10× DPBS, 0.23% 1N NaOH, and H_2_O) to generate the 2% collagen gel. MEFs were collected in a tube and centrifuged at 1000 rpm for 5 min at 4 °C. The cells were resuspended with 2% collagen gel at the density of 1 × 10^6^/mL. The cell mix were seeded into a 48 well and incubated in a cell incubator for 30 min. Appropriate volume medium was added into the wells. The gels were isolated from the well and treated with DMSO/CK-666 for 5 h. The gels were imaged using a camera (Nikon, Tokyo, Japan), and the gel area was calculated by image J.

### 2.12. Stable Cell-Line Generation

Three plasmid-packing systems were used for lentivirus packing. The three plasmids are pLVX-EGFP-N1 inserted with the target genes, ps-PAX2, and pCMV-VSV-G. The packing cell line is HEK293T, and the lentivirus was harvested 48 h after plasmid transfection. Fresh lentivirus-containing media were used to infect Hela cells 3 times until we can see the expression of EGFP. Positive cells were selected by puromycin (Sigma-Aldrich, P8833, Saint Louis, MO, USA) at 2 μg/mL for 1 week.

### 2.13. Statistical Analysis

Statistical differences between two groups of data were analyzed with a two-tailed unpaired Student’s *t* test. All graphical data are represented as a mean with data bars representing SEM.

### 2.14. Polyacrylamide (PA) Gel Preparation and Functionalization

PA gel preparation and functionalization have been adapted from Knoll S.G. et al. [24]. Specially, 40% acrylamide and 2% bis-acrylamide solutions were combined at 8.8% and 0.1%, respectively, to make a 13,800 Pa gel. To achieve a good coverage of fluorescent beads on the PA gel, 100-nm red (594/620) fluorescent beads (Invitrogen, Carlsbad, CA, USA) were mixed with the polyacrylamide gel solution at a volume ratio of 1:1000. Polymerization was initiated by adding ammonium persulfate (APS) and tetramethylethylenediamine (TEMED) at volume ratios of 1:10 and 1:20, respectively. Once initiated, a 15-μL gel solution was quickly pipetted between a confocal dish and a coverslip, forming a sandwich. The cover slip was treated with bind-silane (BioDee, Beijing, China) followed by treatment with 0.5% glutaraldehyde (Sangon Biotech, Beijing, China) solution. This results in the silanisation of the glass surface, which forms a covalent link with the polymerizing gel, assuring firm attachment of the underside of the gel to the coverslip. Once the sandwich was formed, the PA gel was allowed to polymerize for 15 min at room temperature. Once complete, the upper coverslip was peeled off the gel, leaving a thin layer of gel on the activated surface. The PA gel was then washed extensively in phosphate-buffered saline (PBS). The PA gel was functionalized with 2 mL soak solution (137 mM NaCl and 5% (*v*/*v*) glycerol) for 1 h at room temperature. After that, 2 mL of a mixed buffer of 1-ethyl-3-[3-dimethylaminopropyl] carbodiimide hydrochloride (EDC), N-hydroxysulfosuccinimide (NHS), and 2-(N-morpholino) ethanesulfonic acid (MES) (10% (*v*/*v*) glycerol and pH 4.5) in H_2_O were added at concentrations of 15 mM, 25 mM, and 100 mM, respectively. The PA gel was incubated with the mixed buffer in the dark at room temperature for 30 min. Then, the PA gel was coated with fibronectin (50 μg/mL) in phosphate-buffered saline (PBS) for 35 min at room temperature. The PA gel was then washed with PBS three times and stored at 4 °C for up to 2 weeks.

### 2.15. Traction Force Microscopy (TFM) Assay and Data Analysis

TFM assay were conducted using a spinning disk confocal microscope (Dragonfly, Andor; Belfast, UK) equipped with a 63×/1.40 NA oil immersion objective lens. MEFs were incubated with CellTracker^TM^ Green (Invitrogen, 1:1000 dilution, 1 mM, Carlsbad, CA, USA) at 37 °C for 30 min the day before the experiment. Cells were seeded on fibronectin (FN)-coated polyacrylamide gel 8 h before the experiment. Live-cell imaging of MEFs treated with DMSO or CK-666 was taken for 15 min per cell, 1 min per frame. Both FluoSphereRed (561 nm) and green fluorescent protein (GFP) (488 nm) channels were imaged. Temperature control was maintained on the microscope stage using a live-cell chamber. After imaging, cells were perfused with 3 mL of 0.5% trypsin to release cells from the FN-coupled polyacrylamide (PA) substrate and an image of the unstrained substrate was taken in the 488 and 561 channels.

Once traction force maps and fluorescent images were acquired, average traction force magnitude exerted by the whole cell can be calculated. Firstly, cell contour was extracted by built-in morphological functions in MATLAB (R2017a). Then, cell contour was mapped onto traction force maps and set traction forces outside as zero. Finally, average traction force by the cell can be calculated by

$$T_{aver}=\frac{\sum \left|T_{i}\right|}{S}$$

where $\left|T_{i}\right|$ indicates traction force magnitude per pixel and S represents the total area of cell contour. Stress ratio is analyzed by comparison of the before-to-after traction stresses for application of 0.5% trypsin.

## 3. Results

### 3.1. Arp2/3 Inhibition Decreases Active RhoA

**Figure 1:**
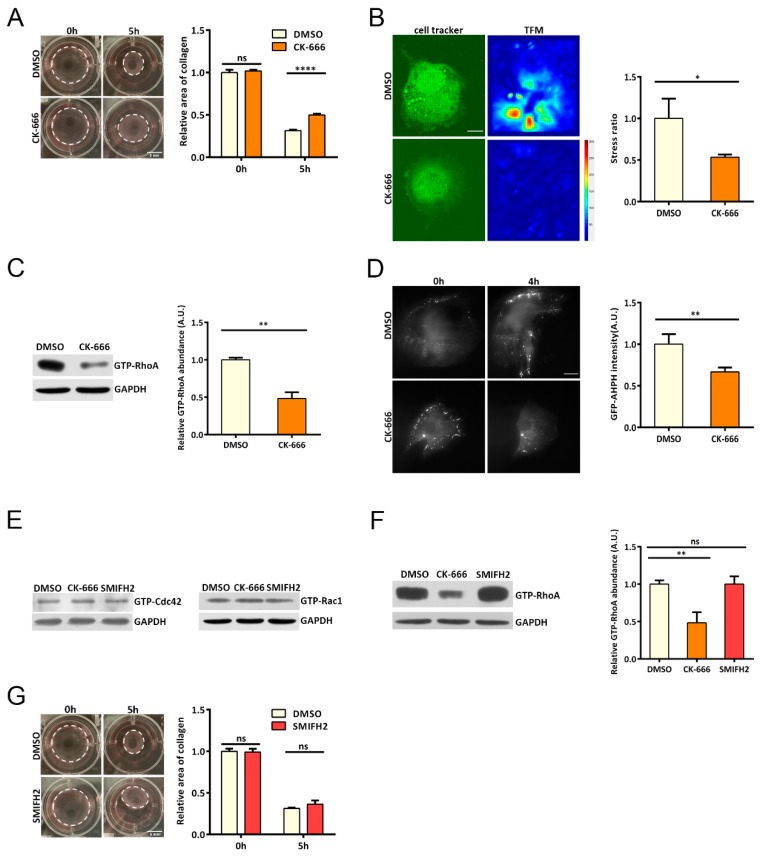
Arp2/3-branched actin maintains an active pool of the small GTPase RhoA. (**A**) Images showing gel deformation at 0 h or 5 h: Mouse embryonic fibroblasts (MEFs) were seeded in 1% collagen gel as indicated, and cells were treated with DMSO or the Arp2/3 inhibitor CK-666. Scale bar: 5 mm, error bar indicates SEM, n = 3 independent experiments. **** *p* < 0.05, by student’s *t* test. (**B**) Traction force microscopy (TFM) images showing beads displacement driven by mouse embryonic fibroblasts (MEFs) treated with DMSO or CK-666. Scale bar is 10 μm, n_DMSO_ = 4, n_CK-666_ = 5. * *p* < 0.05, by student’s *t* test. (**C**) Western blot showing GTP-RhoA level in MEFs treated with DMSO or CK-666 for 5 h: GAPDH was used as loading control, error bar indicates SEM, and n = 3 independent experiments. (**D**) Representative images of MEFs expressing GFP-AHPH treated with DMSO or CK-666 for 0 or 4 h: scale bar is 10 μm, error bar indicates SEM, and n > 6 cells, ** *p* < 0.05. (**E**) Western blot showing GTP-Rac1 and GTP-Cdc42 levels in MEFs treated with DMSO or CK-666 for 5 h. (**F**) Western blot showing GTP-RhoA in MEFs treated with DMSO or SMIFH2: error bar indicates SEM, n = 3 independent experiments, and ns is no significant difference. (**G**) Images showing gel deformation at 0 h or 5 h. MEFs were seeded in 1% collagen gel as indicated, and cells were treated with DMSO or the formin inhibitor SMIFH2. Scale bar: 5 mm, error bar indicates SEM, n = 3 independent experiments. ns, no significant difference.

Mouse Embryonic Fibroblasts (MEFs) depleted from the Arp2/3 complex or treated with CK-666 showed reduction in contractility, as revealed from the gel deformation assay (Figure 1A). Consistently, traction force microscopy (TFM) revealed that the force between the cell and the substrate decreased upon inhibition of the Arp2/3 complex (Figure 1B). Myosin II is one of the major effectors of GTP-RhoA. GTP-RhoA promotes myosin II activity by elevating the phosphorylation level of the regulatory myosin light chain. Accordingly, myosin II showed reduced phosphorylation at Ser18 and 19 (Figure S1A,B), indicative of low actomyosin contraction and consistent with reduced cellular force. We then sought to explore the upstream signaling effectors resulting in the altered cellular force. Interestingly, when we probed the level of active RhoA, we detected significantly reduced GTP-RhoA upon Arp2/3 inhibition (Figure 1C). The decreased fluorescent signal intensity from a RhoA biosensor [25] also suggested reduction in active RhoA when Arp2/3 was inhibited (Figure 1D). Interestingly, neither Rac1 nor Cdc42 showed changes in their activity in the absence of Arp2/3 activity (Figure 1E). We then asked whether this effect on RhoA activity was specific for the branched actin. Using SMIFH2 to inhibit formins did not reproduce similar cell contractility defects or GTP-RhoA reduction (Figure 1F,G), suggesting that the effects on RhoA activity are specifically induced by disruption of the Arp2/3-branched actin.

### 3.2. p190RhoGAP is Responsible for Reduced RhoA Activity upon Inhibition of the Arp2/3-Branched Actin

Elevated GAP activity and/or decreased GEF activity may contribute to the reduced GTP-RhoA level observed in Arp2/3-branched actin-inhibited or -depleted cells. It has been documented that Arp2/3 inhibition alters Yes associated protein (YAP) activity and that YAP nuclear localization regulates ARHGAP29 [26]. Indeed, we detected decreased nuclear YAP when cells were treated with CK-666 (Figure S2A). Meanwhile, when we depleted ARHGAP29, we detected upregulated RhoA activity (Figure S2C), consistent with its cellular function as a RhoA GAP. However, upon Arp2/3 inhibition, we did not detect significant alteration of total ARHGAP29 (Figure S2B). Moreover, in cells lacking ARHGAP29, we still detected reduced GTP-RhoA upon Arp2/3 inhibition (Figure S2D). These results argued against ARHGAP29 playing a major role in regulating RhoA activity in response to Arp2/3 inhibition.

**Figure 2:**
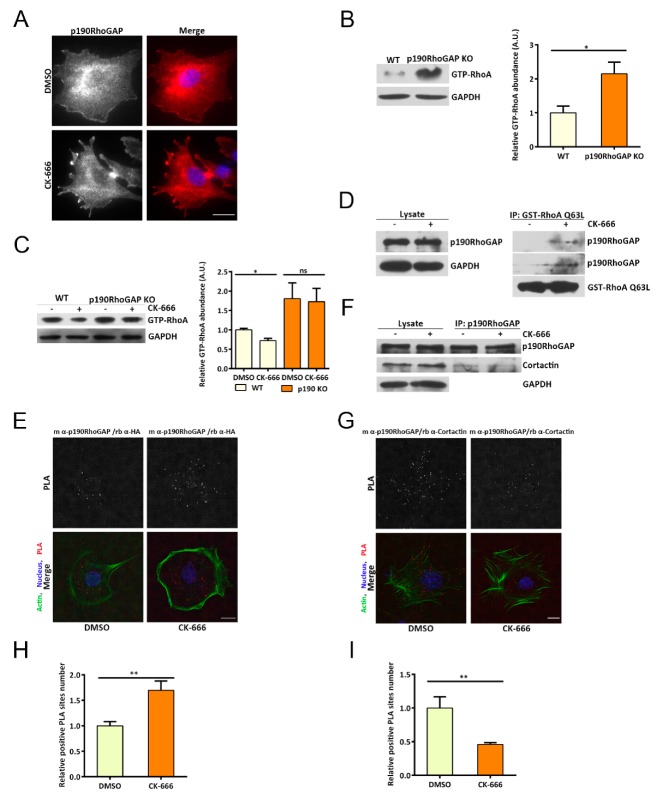
p190RhoGAP is responsible for reduced RhoA activity upon inhibition of the Arp2/3-branched actin. (**A**) Representative immunofluorescent images of p190RhoGAP in MEFs treated with DMSO or CK-666, with red: p190RhoGAP, blue: nucleus, and scale bar of 20 μm. (**B**) Western blot showing GTP-RhoA levels with or without p190RhoGAP: GAPDH was used as loading control, error bar indicates SEM, * *p* < 0.05, by student’s *t* test, n = 3 independent experiments. (**C**) Western blot showing GTP-RhoA in wild-type and p190RhoGAP knockout cell lines treated with DMSO or CK-666: GAPDH was used as loading control, error bar indicates SEM, * *p* < 0.05, ns, no significant difference, by student’s *t* test, and n = 3 independent experiments. (**D**) Western blot showing p190RhoGAP and GST-RhoA Q63L interaction change after DMSO or CK-666 treatment: GST-RhoA Q63L was used to incubate with total cell lysate and then pulled down by GST-conjugated beads. (**E**) Representative images of the proximity ligation assay (PLA; in situ) between p190RhoGAP and HA-RhoA Q63L: the red staining represents positive PLA sites; scale bar is 20 μm. (**F**) Western blot showing p190RhoGAP and cortactin interaction in cells treated with DMSO or CK-666: Co-IP assay was performed as indicated. (**G**) Representative images of the proximity ligation assay (in situ PLA) between p190RhoGAP and cortactin: the red staining represents positive PLA sites; scale bar is 20 μm. (**H**) Quantification of relative positive PLA sites number for the interaction between p190RhoGAP and RhoA Q63L, the error bar indicates SEM, ** *p* < 0.05, by student’s *t* test. (**I**) Quantification of relative positive PLA sites number for the interaction between p190RhoGAP and cortactin, the error bar indicates SEM, ** *p* < 0.05, by student’s *t* test.

We next investigated whether the activity of p190RhoGAP was altered and whether that could explain the changes in GTP-RhoA upon Arp2/3 inhibition. We generated p190RhoGAP knockout cells using the CRISPR-Cas9 system. These cells exhibited significantly higher levels of GTP-RhoA than their wild type counterparts, consistent with the role of p190RhoGAP in decreasing active RhoA (Figure 2B). Interestingly, in these cells, we no longer detected RhoA activity reduction when CK-666 was applied to inhibit the Arp2/3-branched actin (Figure 2C), indicating that p190RhoGAP may mediate the GTP-RhoA regulation upon disruption of the branched actin. If this was the case, loss of Arp2/3 activity may lead to enhanced p190RhoGAP activity or may promote p190RhoGAP-RhoA interaction. Using the active RhoGAP pulldown assay, we indeed observed more GTP-RhoA bound by p190RhoGAP when cells were treated with CK-666 (Figure 2D). Meanwhile, we used the Proximity Ligation Assay (PLA) to confirm the interaction between p190RhoGAP and the constitutively active form of RhoA (RhoA Q63L). We used primary antibodies against p190RhoGAP and RhoA Q63L, followed by oligonucleotide-labeled secondary antibodies binding to primary antibodies. PCR-based amplification reactions then took place where two connector oligos were in close proximity to each other. Positive PLA spots therefore indicated two proteins interacting with each other. We detected increased number of positive PLA sites between p190RhoGAP and RhoA Q63L under Arp2/3 complex inhibition (Figure 2E,H and Figure S2E). These results indicate that the interaction between p190RhoGAP and GTP-RhoA is enhanced upon Arp2/3 inhibition.

The protein level of p190RhoGAP did not change after Arp2/3 complex inhibition (Figure S3A). However, we noticed that p190RhoGAP shifted from leading edge localization to a cytoplasmic distribution when Arp2/3 is inhibited (Figure 2A). Cortactin has been reported to target p190RhoGAP to the cell periphery and to inhibit its activity [15]. Using Co-IP and PLA assays, we found that inhibiting the Arp2/3 complex led to reduced cortactin/p190RhoGAP interaction (Figure 2F,G,I and Figure S2F). These indicate that the interaction between cortactin and p190RhoGAP is diminished upon Arp2/3 inhibition. As a result, p190RhoGAP activity against RhoA may be enhanced, leading to reduction in GTP-RhoA. Collectively, these results suggest a competition for p190RhoGAP between cortactin and GTP-RhoA under the regulation of the Arp2/3–branched actin.

### 3.3. RhoA Abundance is Increased under Arp2/3 Perturbation

**Figure 3:**
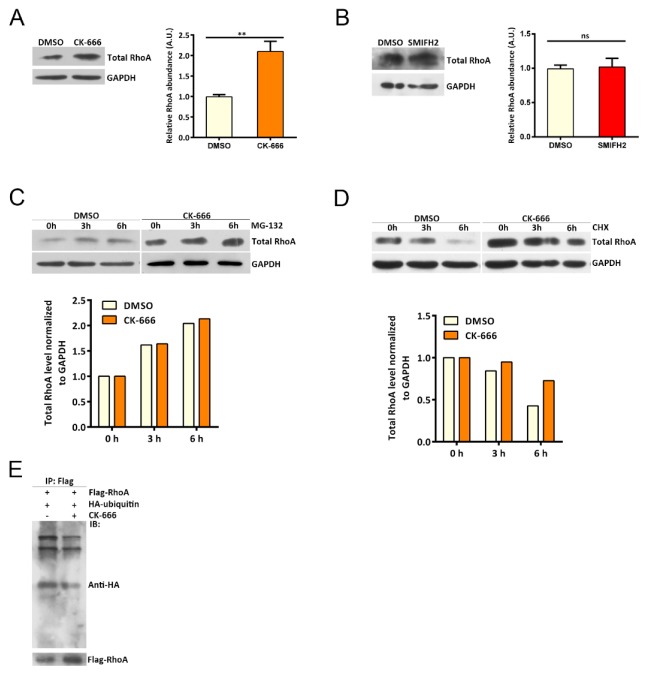
RhoA abundance is increased under Arp2/3 perturbation. (**A**) Western blot showing total RhoA abundance under DMSO or CK-666 treatment: error bar indicates SEM, ** *p* < 0.05, by student’s *t* test. n = 3 independent experiments. (**B**) Western blot showing total RhoA abundance under DMSO or SMIFH2 treatment: error bar indicates SEM, ns, no significant difference, by student’s *t* test. n = 3 independent experiments. (**C**) Western blot showing total RhoA level change in Hela cells treated with MG-132 for 0 h, 3 h, and 6 h with or without CK-666. (**D**) Western blot showing total RhoA level change in Hela Cells treated with cycloheximide for 0 h, 3 h, and 6 h with or without CK-666. (**E**) Western blot showing RhoA ubiquitination level in HEK293T cells under DMSO or CK-666 treatment. HEK293T were transfected with Flag-RhoA and HA-ubiquitin; Co-IP was performed to probe ubiquitinated RhoA.

Another possible mechanism leading to reduced RhoA activity may be the decrease in total RhoA. In that case, less GTP-RhoA will be detected since reduced RhoA protein is present in the cell. However, instead of lowered RhoA levels, we observed significantly increased RhoA protein abundance when CK-666 was applied to cells (Figure 3A). To probe whether the RhoA level alteration is specific to Arp2/3-branched actin perturbation, we applied the formin inhibitor SMIFH2 to cells. Under this treatment, we did not detect obvious changes in RhoA level (Figure 3B). Enhanced synthesis and/or decreased degradation may result in increased protein abundance. To examine whether RhoA synthesis was altered upon Arp2/3 inhibition, we applied the proteasome inhibitor MG-132 to prevent protein degradation. With proteasome inhibition, we detected similar RhoA protein levels between control and CK-666-treated cells at different time points, indicating that branched actin perturbation may not change RhoA synthesis (Figure 3C). To interrogate whether RhoA degradation was hampered upon Arp2/3 defection, we used cycloheximide to inhibit protein synthesis. Interestingly, we found that the drop of RhoA level lagged in CK-666-treated cells compared to controls (Figure 3D). These results indicate that the degradation of RhoA slows down after Arp2/3 inhibition. Consistently, we found that the ubiquitin level of RhoA decreased after Arp2/3 inhibition (Figure 3E).

### 3.4. CCM2 and Smurf1 Involved in Increased RhoA Level under Arp2/3 Perturbation

**Figure 4:**
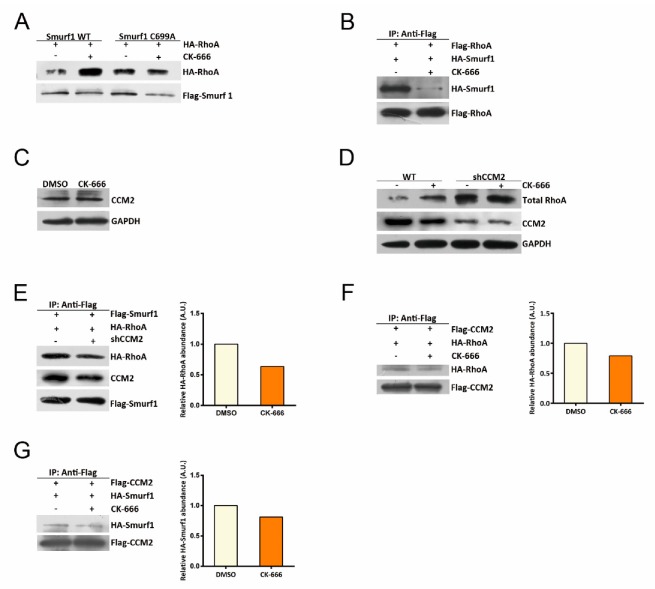
CCM2 and smurf1 are involved in increased RhoA level under Arp2/3 perturbation. (**A**) Western blot showing RhoA level in HEK293T cells overexpressed with smurf1 WT or smurf1 C699A under DMSO or CK-666 treatment for 5 h. CK-666: 100 μM. RhoA abundance is analyzed by immunoblot using anti-HA antibody. (**B**) Western blot showing Smurf1 or RhoA interaction in HEK293T cells transfected with both Flag-RhoA and HA-Smurf1. Co-IP was performed to pulldown Flag-RhoA under DMSO or CK-666 treatment. CK-666: 100 μM. The presence of smurf1 is analyzed by immunoblot using anti-HA antibody. (**C**) Western blot showing CCM2 level in Hela under DMSO and CK-666 treatment for 5 h, CK-666: 100 μM. CCM2 is analyzed by immunoblot using anti-CCM2 antibody. (**D**) Western blot showing RhoA level in both wild-type and CCM2 knockdown HEK293T cells under DMSO or CK-666 treatment. CK-666: 100 μM. GAPDH was used as loading control. (**E**) Western blot showing CCM2 and RhoA interaction changes in HEK293T cells and HEK293T shCCM2 cells, cells were transfected with both Flag-smurf1 and HA-RhoA, and Co-IP was performed to pulldown Flag-Smurf1. The presence of RhoA is analyzed by immunoblot using anti-HA antibody. (**F**) Western blot showing CCM2 and RhoA interaction in HEK293T cells, which transfected with Flag-CCM2 and HA-RhoA: Co-IP was performed to pulldown Flag-CCM2 under DMSO or CK-666 treatment. CK-666: 100 μM. The presence of RhoA was analyzed by immunoblot using anti-HA antibody. (**G**) Western blot showing CCM2 and Smurf1 interaction in HEK293T cells, which transfected with Flag-CCM2 and HA-Smurf1. Co-IP was performed to pulldown Flag-CCM2 under DMSO or CK-666 treatment. CK-666: 100 μM. The presence of Smurf1 was analyzed by immunoblot using anti-HA antibody.

RhoA is degraded through the proteasomal degradation pathway mediated by Smurf1 via the adaptor protein CCM2 [19]. Firstly, we asked whether the increased RhoA abundance under Arp2/3 inhibition was dependent on Smurf1. We overexpressed constitutively inactive Smurf1 (C699A) and found that RhoA abundance no longer increased under CK-666 treatment (Figure 4A). Meanwhile, expressing the wild-type Smurf1 did not abolish RhoA increase upon Arp2/3 inhibition (Figure 4A). These results suggest that Smurf1 plays an important role in mediating RhoA increase induced by Arp2/3 inhibition. Next, we asked whether the interaction between Smurf1 and RhoA was enhanced when cells were deficient in branched actin. By Co-IP assay, we indeed observed that the binding between Smurf1 and RhoA was decreased upon Arp2/3 inhibition (Figure 4B).

CCM2 interacts with both RhoA and Smurf1 [27]. Using the Co-IP system, we first confirmed the interaction among CCM2, Smurf1, and RhoA (Figure 4E). Next, we asked whether CCM2 abundance was disturbed upon inhibition of the Arp2/3 complex. By western blotting, we did not detect altered CCM2 levels upon inhibition of the Arp2/3 complex (Figure 4C). However, when cells were deprived of CCM2, they showed increased RhoA levels and, more importantly, they could no longer further increase RhoA abundance when CK-666 was applied (Figure 4D). These results suggest that CCM2 is involved in Smurf1-mediated RhoA degradation in response to Arp2/3 inhibition.

The interaction between Smurf1 and RhoA decreased in CCM2 knockdown cells compared to control cells (Figure 4E), indicating that CCM2 may facilitate Smurf1 targeting to RhoA. Interestingly, both CCM2-Smurf1 and CCM2-RhoA interactions decreased upon Arp2/3 inhibition (Figure 4F,G). Of note, inhibition of Arp2/3 did not change the protein abundance of Rac1 and Cdc42—two other small GTPases upstream of actin polymerization (Figure S3B,C). Our observations reveal a novel and specific cross talk from the Arp2/3-branched actin to the protein abundance of RhoA, likely through the regulation of CCM2-Smurf1.

### 3.5. Increasing RhoA Activity Rescues Cytokinesis Defects Induced by Arp2/3 Inhibition

**Figure 5:**
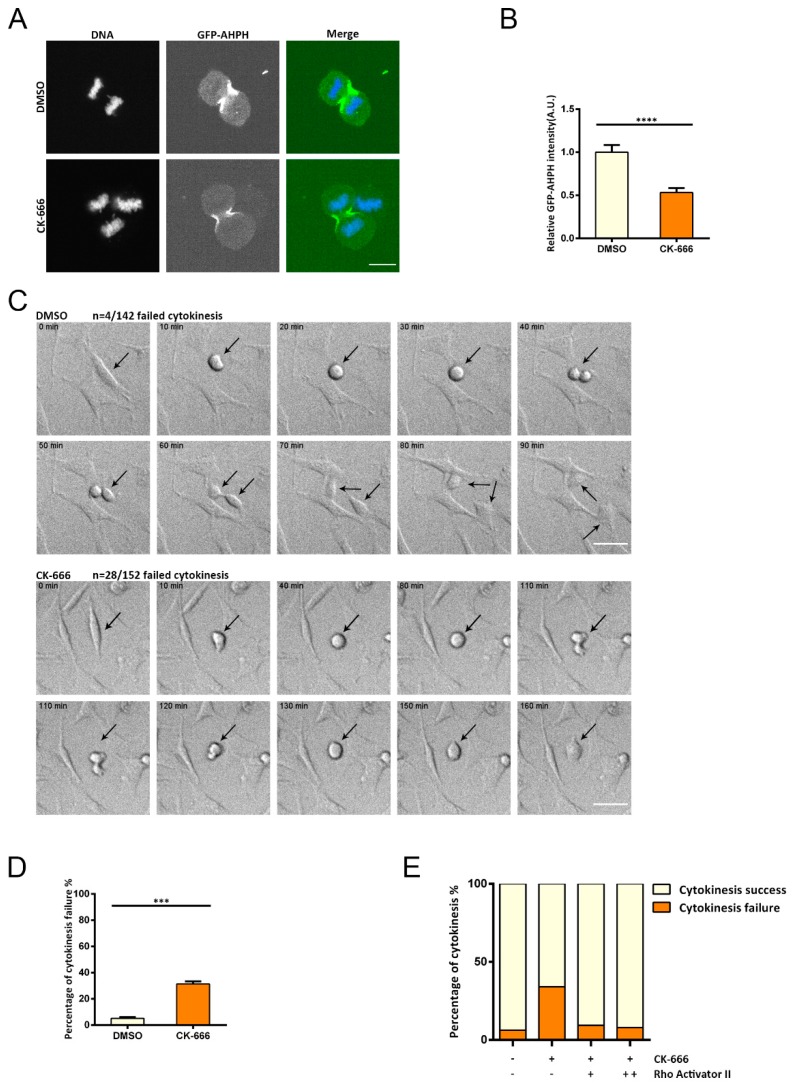
Increasing RhoA activity rescues cytokinesis defects induced by Arp2/3 inhibition. (**A**) Representative images of cells expressing GFP-AHPH in cytokinesis: DMSO or CK-666 were used. Scale bar is 10 μm. (**B**) Quantification of the GFP-AHPH level at the cytokinetic furrow: cells were treated with DMSO or CK-666. n = 13, **** *p* < 0.05 by student’s *t* test. (**C**) Differential interference contrast (DIC) live imaging of DMSO- or CK-666-treated MEFs. The top two panels represent DMSO-treated cells, and the bottom two panels represent CK-666-treated cells. Arrows indicate single cell divisions after drug treatment; 142 cells treated with DMSO and 152 cells treated with CK-666 were scored, and the number of cytokinesis failure/total number of cytokinesis was quantified. Scale bar is 50 μm. (**D**) Quantification of percentage of cytokinesis failure treated with DMSO and CK-666. The error bar indicates SEM, *** *p* < 0.05, by student’s *t* test. n = 3 independent experiments. (**E**) Quantification of percentage of cytokinesis failure treated with DMSO, CK-666, CK-666 + 2 μg/mL Rho activator II, and CK-666 + 4 μg/mL Rho activator II.

RhoA regulates the assembly of the cytokinetic furrow [28]. In order to directly visualize the distribution of endogenous GTP-RhoA, we used a RhoA biosensor (GFP-AHPH), which contains Rhotekin binding domain (RBD) and directly binds to GTP-RhoA [25]. By using this RhoA biosensor, we confirmed the enrichment of GTP-RhoA at the cytokinetic furrow (Figure 5A). It should be noted that this RhoA biosensor is likely to compete with endogenous effectors of RhoA and that the stability and affinity towards RhoA are not optimal [29,30,31]. However, here, we were comparing the difference between control group and Arp2/3 inhibited cells; thus, GFP-AHPH is an available and acceptable way to visualize the distribution of GTP-RhoA. Then, we wanted to investigate whether this ring of GTP-RhoA was re-disturbed upon inhibition of Arp2/3. We performed immunofluorescence to capture the active RhoA signal during cell division in DMSO- or CK-666-treated cells. We quantified the intensity of RhoA biosensor at the cytokinetic furrow. Significant reduction in active RhoA signal was detected at the contractile ring in the absence of Arp2/3 activity (Figure 5A,B).

Meanwhile, we quantified cytokinesis failure in DMSO- or CK-666-treated cells. Our results revealed that Arp2/3-inhibited cells exhibited significantly higher rates of cytokinesis failure (Figure 5C,D and Movies S1 and S2). To test whether the defects in cytokinesis upon Arp2/3 inhibition was linked to RhoA activity change, we used Rho Activator II to increase RhoA activity in combination with CK-666 treatment. We found that the increase in cytokinesis failure under Arp2/3 inhibition can be rescued (Figure 5E). These observations indicate that the Arp2/3 complex may influence cytokinesis through regulation of RhoA activity.

In this study, we have investigated potential feedback regulation from the cytoskeleton to the small GTPases. We identify reduced RhoA activity but increased RhoA abundance upon inhibiting the Arp2/3-branched actin and unveiled altered cortactin/p190RhoGAP and CCM2/Smurf1 interactions to underly these changes. Moreover, we find that Arp2/3 inhibition leads to cytokinesis defects and that this phenotype can be rescued by activating RhoA. Our work highlights the role of Arp2/3-branched actin mediating cellular signal transduction. Whether other forms of cytoskeletal components serve as signaling hubs aside from their fundamental role in maintaining cell morphology and motility remains to be explored.

## 4. Discussion

In this study, we investigated potential feedback regulation from the cytoskeleton to small GTPases. We identified reduced RhoA activity but increased RhoA abundance upon inhibiting the Arp2/3-branched actin and unveiled altered cortactin/p190RhoGAP and CCM2/Smurf1 interactions to underly these changes. Moreover, we found that Arp2/3 inhibition leads to cytokinesis defects and that this phenotype can be rescued by activating RhoA. Our work highlights the role of Arp2/3-branched actin mediating cellular signal transduction. Whether other forms of cytoskeletal components serve as signaling hubs aside from their fundamental role in maintaining cell morphology and motility remains to be explored.

Disruption of the Arp2/3-branched actin leads to reduced GTP-RhoA while increases RhoA abundance; as a net result, there would be increased GDP-RhoA residing in the cell. How the enrichment of GDP-RhoA would affect cellular functions remains an intriguing question to explore. It has been noted that the alteration of certain small GTPase may influence the homeostasis of the GTPases network through Rho-GDI [32]. In our system, inhibition of the Arp2/3 complex by CK-666 did not change the protein abundance or activity level of either Rac1 or Cdc42. However, whether other small GTPase were indirectly affected is unknown and requires systematic studies to be unveiled.

It has been reported that Profilin-1 serves as a gatekeeper for actin assembly by Arp2/3-dependent and -independent pathways [33,34]. Upon depletion or inhibition of the Arp2/3 complex, the liner actin filaments nucleated by formins might enhance. In *C. elegans* embryos, both Arp2/3 RNAi depletion or CK-666 treatment led to cytokinesis delays, which was proposed to be regulated by increases in the cortical and contractile ring levels of both F-actin and the diaphanous formin CYK-1 [35]. In *C. elegans*, inhibition of RGA-5—the homolog of p190RhoGAP—did not cause mitotic defects in *C. elegans* [36,37], while p190RhoGAP is required for proper cytokinesis in Hela cells [28]. This indicates that the function of RGA-5 in *C. elegans* is not the same compared with p190RhoGAP in mammalian cells. Whether inhibition of the Arp2/3 complex in *C. elegans* may cause similar RhoA activity changes though remains an intriguing question to be investigated.

Here, we reported the role of Arp2/3-branched actin in mediating cellular signal transduction. Many factors regulating actin dynamics and Arp2/3 activity have been reported to show altered expression in cancer [38,39,40]. There may exist the possibility that changes in the actin cytoskeleton would affect RhoA abundance and/or activity. Since RhoA is critical for multiple cellular functions such as cell motility and division, whether this regulation existed in cancer would be highly informative in understanding the significance of cytoskeleton and signal transduction in human disease.

## Supplementary Materials

The following are available online at https://www.mdpi.com/2073-4409/8/10/1264/s1, Movie S1. Cytokinesis under DMSO treatment time-lapse movie of MEFs in cytokinesis under DMSO treatment. Time interval: 5 min, total time: 10 h, scale bar: 50 μm. Movie S2. Cytokinesis under CK-666 treatment time-lapse movie of MEFs in cytokinesis under CK-666 treatment. Time interval: 5 min, total time: 10 h, scale bar: 50 μm. Figure S1: pMLC abundance decreases in the absence of Arp2/3 complex. Figure S2: YAP-ARHGAP29 regulates RhoA activity. Figure S3: The abundance of Rac1 & Cdc42 did not change after CK-666 & SMIFH2 treatment.

## Abbreviations

RBD: Rhotekin binding domain; PAK: p21 activated kinase 1 protein; GST: Glutathione S-transferase; p-MLC: phospho-myosin light chain.
