# Supplementary material for: Arp2/3-Branched Actin Maintains an Active Pool of GTP-RhoA and Controls RhoA Abundance
Source: Cells. 2019 Oct 16;8(10):1264. doi: 10.3390/cells8101264 (PMC6830327; doi:10.3390/cells8101264)
Supplement: Supplementary file 1 [file cells-08-01264-s001.zip › cells-594432-supplementary/cells-594432-supplementary.pdf]

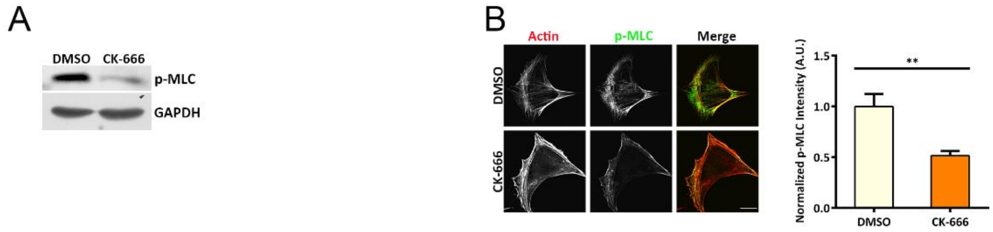

Figure S1. **pMLC abundance decreases in the absence of Arp2/3 complex.**

(A) Western blot showing pMLC level in MEFs treated with DMSO or CK-666. (B) Representative immunofluorescent images of pMLC in MEFs treated with DMSO or CK-666, Scale bar is 20 μm. error bar indicates SEM, n=3 independent experiments. **, P<0. 05.

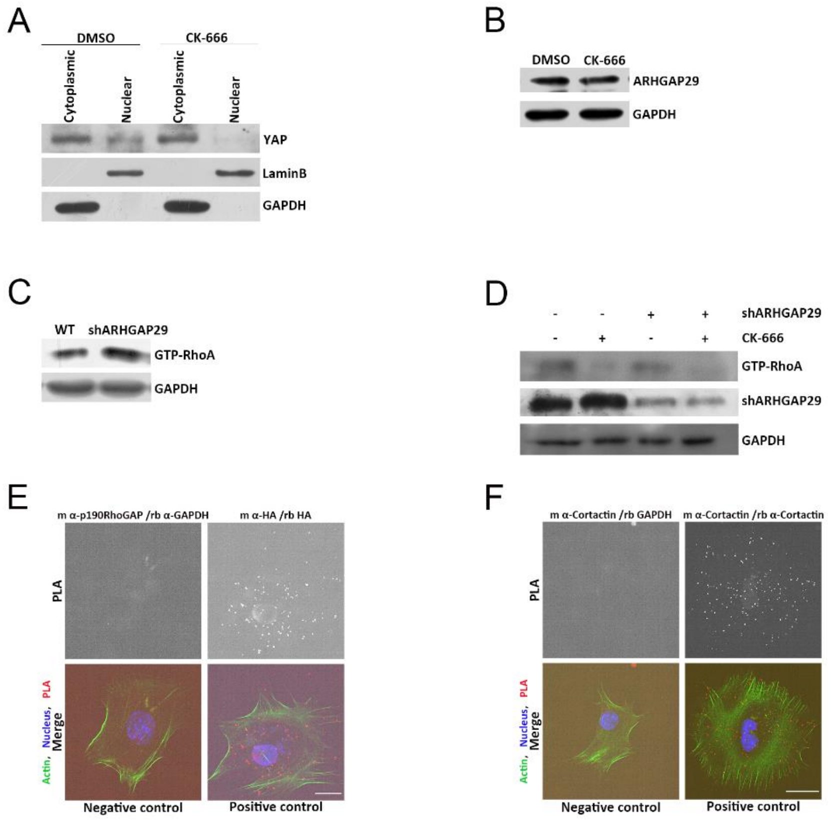

Figure S2. **YAP-ARHGAP29 regulates RhoA activity.**

(A) Western blot showing YAP in the nucleus and in the cytoplasm of MEFs treated with DMSO or CK-666. GAPDH & lamin B were used as loading control. (B) Western blot showing ARHGAP29 level in MEFs treated with DMSO or CK-666. GAPDH was used as loading control. (C) Western blot showing GTP-RhoA level in wild type Hela cells and shARHGAP29 Hela cells. GAPDH was used as loading control. (D) Western blot showing GTP-RhoA level in wild type Hela cells and shARHGAP29 Hela cells treated with DMSO or CK-666. (E) Representative images of the proximity ligation assay (in situ PLA) between p190RhoGAP and HA-RhoA, p190RhoGAP-GAPDH interaction was used as negative control, the red staining represents positive PLA sites,

scale bar is 20 μm. (F) Representative images of the proximity ligation assay (in situ PLA) between p190RhoGAP and cortactin, the red staining represents positive PLA sites, scale bar is 20 μm.

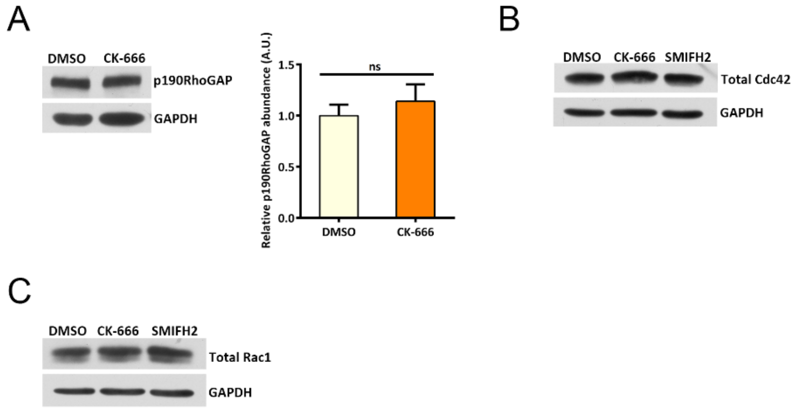

Figure S3. **The abundance of Rac1 & Cdc42 did not change after CK-666 & SMIFH2 treatment.**

(A) Western blot showing p190RhoGAP level in Hela cells treated with DMSO or CK-666. error bar indicates SEM, ns, no significant difference, by student's *t* test. n=3 independent experiments. (B) Western blot showing total Cdc42 level in MEFs treated with DMSO or CK-666 or SMIFH2 for 5h. GAPDH was used as loading control. (C) Western blot showing total Rac1 level in MEFs treated with DMSO or CK-666 or SMIFH2 for 5h. GAPDH was used as loading control.
